# Syndrome Evaluation System for Simultaneous Detection of Pathogens Causing Acute Encephalitic Syndrome in India, Part-2: Validation Using Well Characterized Clinical Samples

**DOI:** 10.3389/fneur.2018.01193

**Published:** 2019-01-15

**Authors:** Sunil R. Govekar, Latha P. Lakshman, Vijayalakshmi Reddy, Reeta S. Mani, Anita Mahadevan, Shankar K. Susarla, Anita Desai, Ravi Kumar Venkata Banda, Ravi Vasanthapuram

**Affiliations:** ^1^Research and Development, XCyton Diagnostics Limited, Bangalore, India; ^2^Neuropathology, National Institute of Mental Health and Neurosciences, Bangalore, India; ^3^Department of Neurovirology, National Institute of Mental Health and Neurosciences, Bangalore, India

**Keywords:** acute encephalitis syndrome, simultaneous detection, molecular diagnosis, validation, syndrome evaluation system

## Abstract

Diagnosis of the aetiological agent in case of acute encephalitic syndrome (AES) continues to pose a challenge in clinical practice as a variety of pathogens are known to cause AES. Here, we report the validation of a Syndrome Evaluation System (SES) developed for simultaneous detection of multiple AES pathogens using a well characterized set of Cerebrospinal fluid (CSF) samples. The validation of the SES was carried out in two phases. In the first phase, the SES was validated using 51 CSF samples obtained from autopsy proven cases and 50 samples obtained from apparently healthy individuals undergoing spinal anesthesia for minor surgeries served as “controls.” The SES detected etilogical agent in 48/51 (94.11 %) samples obtained from autopsy proven AES cases while all the 50 CSF samples obtained from “controls” were negative. In the second phase, the SES was validated using well characterized CSF samples obtained from AES patients fulfilling the WHO case definition of AES (Group I; *n* = 207) and samples that were collected from patients with non-infectious neurological disorder (Group II; *n* = 90). All the samples were tested using multiple conventional/serological assays and categorized into various groups. Amongst the AES cases fulfilling WHO case definition, the SES detected AES pathogens in 160/207 (77.29%) cases while conventional serological/molecular assays were able to detect AES pathogens only in 77/207 (37.1%) of cases. Further, in 12/83 CSF samples that were positive by SES and negative by conventional serological/molecular tests, the results were additionally confirmed by sequencing the PCR products to rule out non-specific amplification in the SES. In patients with non-infectious neurological disorders the SES detected latent viruses 12/90 CSF samples. These results indicate that the SES, apart being a rapid, sensitive, specific, and cost-effective method provides the major advantage of simultaneous detection of multiple pathogens using as single specimen of CSF.

## Introduction

Acute encephalitis syndrome (AES) is a major public health problem in India and a variety of pathogens are known to cause AES (1). The clinical presentation of AES, consists of fever, headache, vomiting, altered mental status, and seizures is encountered in all cases of AES irrespective of the causative pathogen. Therefore, rapid identification of the causative organism and early initiation of specific therapy are crucial for patient management. In a retrospective study carried out in San Diego, California (2), it was noted that patients with a positive CSF PCR result for enteroviruses had significantly fewer ancillary tests performed, received I.V antibiotics for less time and had markedly shorter hospital stay. In addition, they noted that a positive enterovirus PCR result was associated with more rapid hospital discharge. Kay-Yin et al. (3) has computed the cost saving up to $2,700 per patient with timely diagnosis of enteroviral infection. There are a few methods available at present for the simultaneous and rapid detection of multiple pathogens (FILMARRAY®Meningitis/Encephalitis (ME) Panel, by Biomerieux, FTlyo Viral meningitis/FTlyo Bacterial meningitis, by FastTrack Diagnostics) and many of the pathogens are not relevant to the Indian context. Moreover, sensitivity of these tests is not adequate. We developed the Syndrome Evaluation System (SES) which is a pathogen specific multiplex nucleic acid amplification system, wherein the amplified products are identified by sequence specific hybridization. SES is designed for the simultaneous detection of 22 pathogens that are known to cause AES in India. The SES detects DNA viruses such as Herpes simplex virus 1 and 2, Cytomegalo virus, Varicella zoster virus, Human Herpes virus-6, John-Cunningham virus, RNA viruses- Measles, Mumps, Nipah, Rubella, Rabies, Chandipura, Enterovirus, Chikungunya, Japanese Encephalitis virus, Dengue and West Nile, bacteria- *Mycobacterium tuberculosis, Streptococcus pneumonia, Neisseria meningitidis, Haemophillus influenza*, fungus-*Cryptococcus neoformans*, and a parasite *Toxoplasma gondii*. Here we report the validation of the SES using well characterized set of CSF samples obtained from proven and suspected cases of AES as well as those obtained from patients with non-infectious neurological disorders and controls.

## Materials and Methods

### Study Design

The validation of the SES was carried out using well characterized coded clinical samples. The samples used in the study were categorized into various groups as enlisted in Table 1. The blinded validation of SES was carried out in two phases. The study was approved by 68th Institutional Ethics Committee of National Institute of Mental Health and Neurosciences (NIMHANS).

**Table 1:** Description of AES samples used in Phase 1 & 2 of the study (*n* = 398).

	**Category of samples**
**PHASE 1**	
1 A(*n* = 51)	CSF samples obtained from autopsy proven AES cases
1 B (*n* = 50)	CSF samples obtained from patients undergoing spinal Anesthesia
**PHASE 2**	
2A (*n* = 77)	CSF samples positive by one or more of the conventional assays
2B (*n* = 130)	CSF samples negative by any of the conventional assays
2C (*n* = 90)	CSF samples obtained from Non-infectious neurological disorders

### Phase I

Cerebrospinal fluid/brain tissues from autopsy confirmed cases of CNS infections (*n* = 51) archived at Human Brain tissue Repository (HBTR), Department of Neuropathology, NIMHANS, Bangalore were used in this study. A tissue biopsy of the frontal cortex frozen at time of autopsy was also available from all the 51 cases, however, the brain biopsy was used only in 3 Rabies cases in which the CSF yielded negative results in SES. The etiological categorization of the autopsy cases presented in Table 2 was based on histopathology, immuno-histochemical staining and/or use of conventional serological/molecular tests. A second group of CSF samples (*n* = 50) collected from “*apparently*” healthy individuals who underwent spinal anesthesia for minor surgeries served as “controls.”

**Table 2:** Details of the confirmatory assays used to characterize autopsy samples.

**Infections**	**Confirmatory tests CSF**	**Tissue**
Tuberculous meningitis	Antimycobacterial antibody/immune complexes Culture for M tuberculosis CSF ZN stain	ZN stain from basal exudates Histopathology for granulomatous meningitis
Toxoplasma encephalitis	CSF—anti toxoplasma antibodies, PCR for T gondii	Histopathology of brain lesions with immunohistochemical demonstration of tachyzotes of T gondii
Cryptoccoal meningitis	CSF—cryptococcal antigen, India ink and culture confirmation for C. neoformans	Demonstration of budding yeast forms in brain tissue (Periodic acid Schiff and gomori methenamine silver stains)
Rabies encephalitis	CSF—anti rabies antibodies	Fresh brain tissues—fluorescent antibody testing for Rabies Antigen Fixed brain tissues—Negri bodies and immunohistochemical staining for rabies antigen
HSV encephalitis	CSF—HSV antibodies	Histopathology for presence of viral inclusions and HSV antigen by immunohistochemistry in affected areas
Progressive multifocal encephalitis		Histopathology for presence of viral inclusions and JC virus antigen by immunohistochemistry in lesions

### Phase II

The second phase of the validation was conducted using a well characterized panel of CSF samples (*n* = 207) available with Dept. of Neurovirology, NIMHANS. All these samples were obtained between 2007 and 2010 from patients admitted at NIMHANS and who fulfilled the World Health Organization (4) definition of AES. The CSF was divided into two aliquots, one for carrying out conventional virological/serological/molecular assays such as virus isolation/ bacterial culture, serological/molecular diagnostic assays detailed in Table 3. In addition, another set of CSF samples (*n* = 90) obtained from non-infectious neurological conditions comprising of head injury (*n* = 31), brain tumors (*n* = 22), epilepsy (*n* = 2), Guillain Barre's syndrome (*n* = 11), and demyelinating disorder (*n* = 24) was also tested as controls.

**Table 3:** Details of the Conventional assays used to classify the CSF samples into various groups.

**Pathogen**	**Conventional assay used**	**Source**
Herpes Simplex Virus	RT-PCR	Professional Biotech^*^, India
Cytomegalovirus	RT-PCR	Professional Biotech^*^,India
Varicella zoster virus	RT-PCR	Professional Biotech^*^,India
HHV-6	RT-PCR	Professional Biotech^*^, India
M. tuberculosis	Culture of CSF	Department of Neuromicrobiology, NIMHANS
T.gondii	Antibodies to T.gondii in CSF by Latex agglutination test	Plasmatech^#^
C. neoformans	1. India Ink staining on CSF, 2. Culture from CSF	Department of Neuromicrobiology, NIMHANS
S. pneumoniae	1. Culture 2. CDC, USA, RT-PCR	Dept. of Neuromicrobiology Dept. of Neurovirology
N. meningitides	1. Culture 2. CDC, USA, RT-PCR	Dept. of Neuromicrobiology Dept. of Neurovirology
H. influenzae	1. Culture 2. CDC, USA, RT-PCR	Dept. of Neuromicrobiology Dept. of Neurovirology
Measles	RT-PCR	Professional Biotech^*^, India In House ELISA
Rubella	RT-PCR	Professional Biotech^*^, India
JEV	1. IgM Capture ELISA 2. Virus Isolation 3. CDC approved TaqMan RT PCR	1. JEV CheX, XCyton Diagnostics Pvt. Ltd., 2. C6/36 Cell line 3. Dept. of Neurovirology
Enteroviridae	RT-PCR	Professional Biotech^*^, India
Chikungunya	1. RT-PCR 2. IgM Capture ELISA	Professional Biotech^*^, IndiaNIV, Pune
Dengue	IgM Capture ELISA	NIV, Pune

* *Professional Biotech, India, is a commercial source which supplies ready to use CE marked Real Time-PCR kits from Corbett, Life sciences*.

# *Plasmatech, is a commercial source which supplies Latex Agglutination test for the detection of antibodies to Toxoplasma gondii*.

### Nucleic Acid Extraction

Nucleic acids were extracted from 140/200 μl of CSF sample using commercial columns (Qiagen, USA) as per the procedure specified in the instruction manual provided by the manufacturer.

#### cDNA Synthesis

Reverse transcription of total RNA extracted from the CSF samples were carried out using a commercial cDNA Archive Kit (ABI, USA). cDNA was synthesized at 45°C for 30 min in a final volume of 50 μl, using standardized concentration of pathogen specific primers, 25 μl of RNA, 2 μl Multi-Scribe reverse transcriptase (50 Units/μl), 2 μl of 25 × dNTP mix, 5 μl of 10 × RT buffer and 1 μl of RNAse inhibitor.

#### Nucleic Acid Amplification

Nucleic acid amplification was standardized in a 50 μl volume containing 4 mM magnesium chloride, 0.2 mM deoxynucleoside triphosphates, standardized concentration of each primer set in at 50 to 300 nM concentration and 1 U of Taq polymerase (ABI, USA). For each sample two multiplex amplifications were carried out, one for all DNA pathogens and the other RNA pathogens using the cDNA. The initial denaturation step was carried out at 95°C for 10 min followed by 40 cycles of denaturation at 95°C for 45 s each, annealing at 60°C for 45 s and extension at 72°C for 45 s in a thermal cycler (*Bio-Rad*, UK). The PCR products were detected by hybridization on the SES platform.

#### Hybridization

Signature gene sequences chosen as probes for each of the pathogen were commercially synthesized. 20 to 160 μM of probes for each of the pathogen were transferred on to a pre-determined position on the SES platform according to the templates. Two different templates were used, one for identification of DNA pathogens and the other for identification of RNA pathogens. The SES platforms were subsequently baked at 80°C for 20 min to immobilize the probes on to the solid phase of the platform.

Detection of the amplified products was facilitated by using biotin labeled primers. Briefly, hybridization was carried out by heat denaturing the amplified product at 95°C for 10 min. The denatured amplified products were then diluted in the hybridization buffer and transferred on to the SES platform and the platform was incubated at 50°C for 30 min. Unbound amplicons were removed by washing the device thrice with pre-heated wash buffer. Following the washes, conjugate (Streptavidin peroxidase, Thermo) diluted in conjugate buffer containing 1% BSA in PBS along with 0.05% tween 20 was added and incubated for 15 min at room temperature. Unbound conjugate was removed by washing thrice with the conjugate buffer at room temperature. Subsequently freshly prepared substrate (0.5 mgs/ml of Diaminobenzidine, HCl with 0.03% of H_2_O_2_) was added and incubated for 10 min at room temperature. SES platforms were then washed with water and the signal observed with naked eye under adequate illumination. Interpretation results was carried out by two independent observers who were blind to the clinical category of the sample and was aided by the use of a standardized grid described elsewhere (5).

#### Sequencing

Sequencing of the entire length of the nucleic acid amplification products was carried out only in CSF samples that yielded a discrepant result between the conventional assays and the SES. This was facilitated by cloning of the amplified PCR product using the commercially available TOPO-TA cloning kit. Cloning was carried out as per the manufacturer's instruction. The cloned product was then subjected to sequencing using Sanger's method commercially.

## Results

The overall summary of the results obtained in Phase 1 and Phase 2 is presented in Table 4. The SES yielded concordant results in 48/51(94.11%) samples of autopsy confirmed CSF samples. However, it failed to detect viral RNA in 3/10 CSF samples obtained from autopsy confirmed Rabies cases. In these three rabies cases, CSF samples were indeed negative for Rabies viral RNA when tested on the conventional RT-PCR assay as well. Nevertheless, RNA extracted from the frozen brain tissue samples of these three cases was positive for Rabies viral RNA on the SES. Amongst the control group (1B), the SES did not detect any pathogen in the 50 CSF samples which were obtained from people undergoing spinal anesthesia for minor surgeries.

**Table 4:** Summary of results of SES AES Validation.

**Groups**	**No. tested**	**SES positive**	**SES negative**	**Total**
Autopsy proven sample (Phase 1)	51	48	3^*^	51^**^
Normal (Phase 1)	50	–	50	50
AES positives by conventional assay (Phase 2)	77	77	–	77**^#^**
AES conventional assay negative (Phase 2)	130	83**^∧^**	47	130
Neurological controls (Phase 2)	90	12	78	90
Total	398	220	178	398

* All the three SES negative samples were autopsy proven Rabies cases

** Detailed breakup of results obtained in Autopsy proven cases provided in Table 5

# Detailed breakup of results obtained in conventional assay positive cases provided in Table 6

∧ *Detailed breakup of results obtained in conventional assay negative cases provided in Table 7*.

The results of the Phase 2 evaluation of the SES is presented in Table 4, revealed that concordant results with the conventional assay systems was obtained in 77/207 CSF samples of AES cases. On the contrary, the SES detected nucleic acid of different pathogens in 83/130(63.8%) CSF samples obtained from AES cases (Group B), all of which were negative by conventional diagnostic assays. Furthermore, among the samples obtained from non-infectious neurological cases (Phase 2C), SES detected pathogens in 12/90 (13%) samples. All these 12 SES positive samples were from obtained from patients undergoing chemotherapy for brain tumors. This included four samples positive for HSV, four for CMV, and two each for HHV-6 and JCV. The four HSV positive samples were further reconfirmed by the conventional RT-PCR assay. However, reconfirmation of CMV, HHV-6, and JCV positive samples could not be carried out due to paucity of CSF samples.

Infections due to multiple pathogens were detected in as many as 30 samples. Amongst these 10 were noted in autopsy proven cases (Table 5, Phase 1A), 8 in AES cases that were positive both by conventional assays and SES (Table 6, Phase 2A) and 12 in AES cases that were negative by conventional assays and positive by SES (Table 7, Phase 2B). In order to rule out non-specific amplification in the SES as well as to confirm the true positivity, 12/83 samples which were negative by all the conventional assays but positive by SES were subjected to DNA sequencing. Toward this a uniplex amplification of the pathogen detected in the SES was carried out followed by purification of the amplicon, cloning of the amplicon, and sequencing of the cloned product. In case of polymicrobials uniplex amplification of both the pathogens was carried out followed by cloning and sequencing. All the 83 samples, positive in Phase 2B could not be confirmed by sequencing due to paucity of CSF sample. Confirmation by sequencing was possible only 12 samples were CSF was available after carrying out all the tests. The result of the sequencing is presented in Table 8. As evident from Table 5, in all the 12 samples where sequencing was carried out to confirm the specificity of the product, the results matched those obtained with SES.

**Table 5:** Detailed breakup of results obtained in Autopsy proven cases (Group 1A).

**Category of sample**	**Organisms detected at autopsy**	**Nos. tested**	**No. positives on SES**	
Autopsy Proven	HSV	06	06	HSV
	JCV	05	03	JCV
			02	JCV+HSV
	M.tuberculosis	07	05	M.tuberculosis
			02	M.tuberculosis+HSV
	C.neoformans	10	04	C.neoformans
			06	C.neoformans+CMV
	T.gondii	13	13	T.gondii
	Rabies	10	07	Rabies
TOTAL		51	48	

**Table 6:** Detailed breakup of results obtained in conventional assay positive cases (Group 2A).

**Category of sample**	**No. tested**	**No. positive on SES**		
C. neoformans (India Ink)	09	09	07	C.neoformans
			02	C.neoformans + S. pneumoniae
TBM(Culture or smear)	16	16		
T. gondii(ELISA)	05	05	03	T.gondii
			01	T.gondii+ CMV
			01	T.gondii +C.neoformans
S.pneumoniae	08	08		
N.meningitidis	06	06	02	N.meningitidis
			04	N.meningitidis + S.pneumoniae
HSV RT PCR	13	13		
Enterovirus RT PCR	11	11		
Chikungunya IgM	07	07		
Measles IgM	01	01		
Dengue IgM	01	01		
TOTAL	77	77		

**Table 7:** Detailed breakup of results obtained in conventional assay negative cases (Group 2B).

**Conventional test negatives**	**SES results**			
*n* = 130	Positives	83		
	HSV	26	21	HSV
			02	HSV +S.pneumoniae
			01	HSV+ JC
			01	HSV+ CMV
			01	HSV+ HHV-6
	CMV	18	15	CMV
			02	CMV+ JC
			01	CMV+ S.pneumonia
	S. pneumoniae	15	14	S.pneumoniae
			01	S.pneumoniae + N.meningitides
	TBM	05	02	TBM
			02	TBM + S.pneumoniae
			01	TBM + VZV
	JC	03		
	Entero	04		
	Chikungunya	02		
	H.influenzae	02		
	N.meningitidis	02		
	Cryptococcus	01		
	HHV-6	01		
	VZV	04		

**Table 8:** Validation of SES results by sequencing of re-amplified PCR products.

**Sl no**	**Identity of sample**	**Phase**	**Conventional assay results**	**SES result**	**Sequencing results**
1	73	2B	Negative	HSV	Concordant with SES
2	12	2B	Negative	HSV	Concordant with SES
3	48	2B	Negative	VZV	Concordant with SES
4	234	2B	Negative	VZV	Concordant with SES
5	246	2B	Negative	VZV	Concordant with SES
6	420	2B	Negative	S.pneumoniae	Concordant with SES
7	996	2B	Negative	S.pneumoniae	Concordant with SES
8	42	2B	Negative	S.pneumoniae	Concordant with SES
9	94	2B	Negative	H.influenzae	Concordant with SES
10	640	2B	Negative	N.meningitidies	Concordant with SES
11	334	2B	Negative	S.pneumonia and N. meningitidies	Concordant with SES
12	59	2B	Negative	HSV and S.pneumoniae	Concordant with SES

## Discussion

The SES described in the study was developed specifically to address the unmet clinical and epidemiological needs of AES cases that occur in India. Although several multiplex systems for detection AES pathogens are available commercially (FILMARRAY®Meningitis/Encephalitis (ME) Panel, by Biomerieux, FTlyo Viral meningitis/FTlyo Bacterial meningitis, by FastTrack Diagnostics) none of them offer multiplexing options for detection of 22 pathogens relevant in the Indian context. In one of the multi-centric study on Film-array ME panel the authors have indeed noted a sensitivity of 32% and specificity of 68% with respect to the bacterial pathogens when compared with regular cultures. Most importantly, *S.pneumoniae*, which is one of the most important pathogen causing AES was wrongly identified in 80% of the cases (6). Indeed, multiple panels of these commercial multiplex PCR assays need to be used to cover the AES pathogens relevant to the Indian context (e.g., JEV, DEN, CHIKV). The SES was validated in two phases. In phase I of this study, the SES was validated using 51 CSF samples obtained from autopsy proven cases of brain infections and 50 CSF samples obtained from apparently healthy “control” subjects undergoing spinal anesthesia for minor surgeries. The results revealed that the SES had an overall sensitivity of 94.1% with samples obtained in autopsy proven cases. The three “*false negatives*” obtained in SES were cases of Rabies which were indeed negative for rabies virus nucleic acid in the CSF samples by the conventional assay as well. This apparent lack of nucleic acid in the CSF may be due to the sequestration of the virus into the cellular compartment of the brain, thus, bringing down the levels of virus in CSF below detection limit. However, these three rabies cases were positive on SES for the presence of rabies RNA when brain biopsy material was used as sample. Therefore, it is likely that these three samples were not true “*false negatives”* on SES. It is well documented that CSF nucleic acid tests give an overall sensitivity of 8–43.3% for rabies diagnosis (7, 8). However, SES has yielded 70% detection of Rabies viral RNA in CSF, which is much higher than any other assay described.

Having established the sensitivity of the SES using samples obtained from autopsy proven cases, the specificity of the SES was assessed using 50 control CSF samples obtained from patients undergoing spinal anesthesia for minor surgical ailments. The negative results obtained in all the 50 samples of this group confirms that the SES is 100% specific. This further negates the general fear that sensitive assays such as nucleic acid detection may yield positive results for latent viruses (9).

In the second phase, the SES was validated using samples that were categorized as AES according to WHO case definition in comparison to conventional assay system used for diagnosis. It is noteworthy that all pathogens positive by the conventional assays were indeed detected as positive by SES (Phase 2A, Table 4). This further confirms the sensitivity of SES in detecting the pathogens with 100% sensitivity.

The main advantage of SES demonstrated in this validation, is its ability to detect pathogens in 83/130 (63.8%) of AES cases which were negative by the conventional assay (Phase 2B, Table 4). This underscores the enhanced sensitivity of the SES in determining the specific etiology of clinically suspected AES cases. It was noteworthy that in 76/83 (91.5%) samples the SES yielded a positive result for pathogens for which, specific therapy is available (Group 2B, Table 7). A shift to appropriate antibiotic therapy was achieved due to higher detection rate of SES (10). Further, due to its higher detection rate SES is proven to reduce mortality in neonatal sepsis by five-fold as well as reduce antibiotic usage and hospital stay in a RCT conducted at JIPMER, Pondicherry (11). This highlights the utility of the SES not only as a diagnostic tool but as a therapeutic guidance system as well. The enhanced sensitivity of the SES is attributable to two factors; firstly, the use of highly specific primers derived from conserved regions of virulent genes and secondly, to the use of biotin-avidin detection system.

In order to further establish the specificity of SES and rule out the possibility of “*false positive*” results obtained in samples that were negative by conventional assays DNA sequencing was carried out. In 12/83 cases where the SES alone yielded positive results and sufficient quantity of CSF was available for further investigations were subjected to DNA sequencing. The sequencing data were identical to the results obtained in SES. This indicates that the positive results obtained in 83/130 cases were indeed true positives. In view of the enhanced sensitivity, SES can be used as a first line diagnostic in a clinical setting where it can lead to better patient outcomes by prompt institution of specific therapy early in the course of illness.

In phase 2 non-infectious neurological samples were used as controls (*n* = 90). Amongst, these 78 samples were negative on SES for all the pathogens while 12 samples were positive for various latent viruses (HSV-4; CMV-4; JC-2, and HHV-6-2). All these 12 samples were obtained from patients who had brain tumors and were on chemotherapy. Reactivation of the latent viruses such as HSV, CMV, HHV-6 is well known among patients whose immune status is compromised following chemotherapy (12–14). Of these, 4 cases of HSV were reconfirmed by the conventional RT PCR assay used in this validation revealing the true status of these samples. Unfortunately, clinical follow up was not available to ascertain if the reactivated virus caused encephalitis in these patients.

The results of this study also highlight that the SES detected pyogenic bacteria in 19 patients where the conventional standard RT PCR assays failed to do so (Table 7). It is well documented that pyogenic bacterial infections can clinically present as AES (15). Higher sensitivity of the SES is particularly invaluable in the pediatric age group where the CSF cytology reports are inconclusive due to empirical antibiotic therapy initiated even before the availability of laboratory reports (16, 17). In these cases, detection of nucleic acids is the only option available for determination of infectious etiology.

Detection of polymicrobial infections is one of the highlights of this study. Initiation of appropriate therapy in patients detected with polymicrobial infections has led to better patient's outcome (18). In 51 CSF samples obtained from autopsy proven cases multiple pathogens (HSV and CMV in addition to the etiological agent detected at autopsy) were identified in 10 CSF samples by SES. All these samples were obtained from HIV infected individuals. Opportunistic infection due to multiple pathogens is a known phenomenon amongst HIV infected individuals (19, 20). In two autopsy proven cases, *M. tuberculosis* was detected by CSF culture 3 months prior to death and treated with anti-tuberculous therapy. On post mortem these two cases were categorized as *M. tuberculosis* based on gross morphology (basal exudates). However, the SES revealed a positive result only for T.gondii nucleic acid in these two cases. The brain tissue of these two cases was therefore subjected to re-examination by neuropathologists. Upon detailed histopathological examination and immunocyto-chemistry it was reconfirmed by the neuropathologists that they were indeed positive only for *T. gondii* and not for *M. tuberculosis*. Such a misidentification of pathogen can be avoided if the classification is carried by nucleic acid detection. It was noteworthy that SES also detected HSV along with *S. pneumonia* in two cases and dual infections due to *S. pneumonia* and *N. meningitides* in four other cases. One each of these two categories of mixed infections was also confirmed by DNA sequencing. Mixed infections of *N. meningitides* and *S. pneumonia* have earlier been documented in literature (21, 22).

In conclusion, the SES validation has revealed that it has 100% sensitivity and specificity in identifying pathogens accurately in comparison with the conventional assay. Moreover, SES has an enhanced sensitivity of 63.8% in detecting the pathogen that are negative by conventional assays. Overall, SES detected 160/207 (77%) AES cases while a battery of conventional assays detected only 77/207 (37%). The main advantage of SES is that detection of multiple pathogens in a single sample that can be achieved in just 7 h using a single assay facilitating rapid and comprehensive diagnosis of AES cases. Apart from enabling institution of early specific therapy in individual cases in clinics, the SES can facilitate public health management in outbreaks of AES in the community.

## Author Contributions

SG, LL, AD, RB, and RV contributed in conception and design of the study. SG performed the testing of all the samples on SES. RM and VR performed the testing and characterization of the clinical samples based on the conventional tests. AM and SS were also involved in concept design and provided pathology proven clinical samples. SG wrote the whole manuscript which was read, revised and approved by RB and RV.

### Conflict of Interest Statement

SG, LL, and RB are employed by XCyton Diagnostics Pvt Ltd. The remaining authors declare that the research was conducted in the absence of any commercial or financial relationships that could be construed as a potential conflict of interest.
